# The Use of Palaeoecology in UK Peatland Restoration: Perspectives From Practice

**DOI:** 10.1002/ece3.74620

**Published:** 2026-11-13

**Authors:** J. Gauld, E. L. Shuttleworth, W. Fletcher, J. Akerman, H. Harper

**Affiliations:** ^1^ Department of Geography, School of Environment Education and Development The University of Manchester Manchester UK; ^2^ IUCN UK Peatland Programme, The Kiln Newark UK

## Abstract

Extensive degradation has necessitated high‐priority restoration of UK peatland ecosystems. Palaeoecology has great potential to support peatland restoration, offering a long‐term perspective of environmental change that can help determine baseline conditions, restoration targets, and intervention success. However, it is suggested by palaeoenvironmental researchers that palaeoecology is underutilised in practical settings, with few examples of applied palaeoecological studies within academic literature. This study uses a practice perspective to evaluate the extent to which palaeoecology is incorporated into UK peatland restoration and identifies barriers and opportunities for integrating long‐term ecological research into restoration practice. A desk‐based study of 68 UK peatland restoration projects and survey of 32 peatland practitioners were used to explore the use of palaeoecological research as part of UK peatland restoration projects. A total of 10 instances of applied palaeoecology in UK peatland restoration were found between the desk‐based study and practitioner survey. The findings indicate that palaeoecology is rarely utilised in UK peatland restoration practice, with limitations in funding, time, and academic input identified as key barriers. Cases where palaeoecology has been considered primarily utilised pollen analysis and radiocarbon dating, with practitioners largely involved in fieldwork and research design. This study makes two recommendations to facilitate the incorporation of palaeoecological research into UK peatland restoration practice: (i) better dissemination and documentation of case studies to provide greater evidence of how palaeoecology may be used to support UK peatland restoration and clearer insight into the use of palaeoecology in practice; (ii) better two‐way knowledge transfer between palaeoecologists and practitioners to identify restoration challenges and knowledge gaps where palaeoecology could contribute.

## Abstract 2

Palaeoecology has the potential to support peatland restoration, but appears to be underutilised in practice. This study uses a desk‐based review and practitioner survey to gain a practice perspective on the current use of palaeoecology in UK peatland restoration practice. The study finds that palaeoecology is rarely utilised in peatland restoration practice and provides practical recommendations to improve application of palaeoecology in peatland restoration.

## 1 Introduction

Peatlands are highly valuable ecosystems that contribute to critical services, such as carbon storage, water provision and quality, biodiversity support, flood risk alleviation, rural economies, recreation, and cultural heritage (McCarroll et al. 2016a; Manton et al. 2021). Yet in the UK, they largely exist in a severely degraded state, evidenced by a dramatic decline in peatland area and condition, decrease in species richness, and over dominance of vascular plants (McCarroll et al. 2016a; Davies 2016). This has wider implications for the state of peatland function and self‐regulation and the ability of peatlands to provide ecosystem services (Davies 2016; McCarroll et al. 2016a, 2016b). The extent of peatland damage has made restorative action a high‐priority focus of policy and research (Whitfield et al. 2011; Andersen et al. 2017), exemplified through the UK Government's *England Peat Action Plan* (DEFRA 2021) and the targeting of peatlands within international conventions like the United Nations' Decade on Ecosystem Restoration 2021–2030 (Tanneberger et al. 2021, https://www.decadeonrestoration.org/what‐decade).

Despite substantial investment into UK peatland restoration, projects have been broadly criticised for lacking specific goals and a common understanding of what successful restoration trajectories and outcomes look like (Andersen et al. 2017; Everson et al. 2026). Targets are often based on relatively recent surveys (such as which comprise the National Vegetation Classification; NVC; Rodwell 1991) or assumed pristine (unmodified or undisturbed) sites, with few cases of long‐term monitoring of pre‐ and post‐restoration peatlands (Lunt et al. 2010; Everson et al. 2026). However, peatlands are diverse, adaptive, and dynamic ecosystems that have been modified for millennia through disturbances (i.e., factors that deviate the ecosystem from its existing conditions) such as drainage, grazing, fire, agriculture, pollution, and industrial or domestic peat cutting (Łuców et al. 2022; Ritson et al. 2025). Moreover, the compounding effects of multiple disturbances can have long‐lasting impacts on ecohydrological processes and peat physical properties, with implications for restoration such as capacity to recover carbon sequestration or recolonise *Sphagnum* (Lunt et al. 2010; Sherwood et al. 2013; Swindles et al. 2016; Word et al. 2022). This extensive history of peatland modification means that contemporary reference points may represent already modified systems, creating uncertainty over their suitability as restoration targets.

Incorporating a longer‐term perspective, such as may be gained from historic vegetation records, into restoration planning can provide a valuable temporal dimension to current methods of setting targets and evaluating restoration progress. Identifying a singular point in time as a historic baseline (i.e., the environmental conditions that existed prior to an event(s) of natural or anthropogenic‐driven change, Jentsch et al. 2022; Guilfoyle et al. 2024) and attempting to return the ecosystem to this point has been criticised as unrepresentative of the dynamic nature of ecological systems and unrealistic in the face of unprecedented anthropogenic and climatic pressures (Łuców et al. 2022). However, ongoing environmental degradation can influence what is considered normal or natural, a phenomenon called ‘shifting baseline syndrome’ (Soga and Gaston 2018) and limiting our perception of desirable ecosystem states to contemporary ecosystems alone risks acceptance of declining conditions (Ritson and Lindsay 2023; Everson et al. 2026). For example, Ritson and Lindsay (2023) demonstrate how examining peatland archival records can support a greater floristic diversity in ecological targets than what is suggested through 20th century vegetation surveys, as more recent ecosystem appraisals can reflect an environment that has already been altered by anthropogenic activities. However, historical accounts are often temporally restricted, as demonstrated in Ritson and Lindsay (2023) where the earliest archival records begin in 1853.

Palaeoecology—the study of past ecological systems using preserved materials such as fossil pollen, leaves, seeds, and insect and other faunal remains—offers an alternative route to gaining a longer‐term perspective of environmental change over hundreds or thousands of years (H. J. B. Birks 2012). The temporal scale of palaeo‐records means that palaeoecology can offer insight into landscape and vegetation change and processes that occur on timescales beyond the reach of contemporary monitoring or historic environmental records (Barak et al. 2016; Siggery et al. 2023). By contextualising contemporary environments within a longer‐term perspective of ecological change and processes, palaeoecology can examine the drivers and trajectories of change and provide evidence that restoration targets are ecologically suitable for the site (such as using historic presence to guide species reintroductions) or are achievable given the site's disturbance history (such as whether legacy impacts are limiting vegetation or hydrological recovery) (Swindles et al. 2016; Taylor et al. 2019). This can enable conservation practitioners and land manager to make decisions with an understanding of how past disturbance events and climatic shifts may have influenced ecosystem composition and stability.

The potential for palaeoecological research to inform peatland restoration practice has been much discussed in recent decades (J. J. B. Birks 1996; Davies et al. 2014; Chambers and Daniell 2011; Clarke and Lynch 2016; Marcisz et al. 2022; McCarroll et al. 2016a, 2016b, 2017). Yet, examples of palaeoecological studies aiming to inform practitioners are relatively uncommon (Davies et al. 2014; Gauld et al. 2025). Rare collaborative efforts between researchers and conservationists serve to illustrate the value of conservation‐focused palaeoecology for defining a baseline of pre‐disturbance activity, determining the longevity of current conditions, and understanding ecosystem resilience or resistance to disturbance. For example, Siggery et al. (2025) co‐designed a palaeoecological study with conservation practitioners to use ecological history to inform management of lowland heathland. Key outcomes included the identification of key species for future monitoring and contextualisation of present‐day fire frequency and vegetation community within the historic context, illustrating more frequent occurrence of fires compared to the site's past. Such studies illustrate how palaeoecology can help to better understand the present‐day site within its historical context and can have direct relevance for site management, such as emphasising rewetting as a tool against wildfire and climate change.

There is growing support from academic and practitioner communities for the inclusion of palaeoecology in restoration planning. However, the impacts of palaeoecological research on practice are not always easily discernible from the academic literature (Bunting et al. 2026), which is often produced to fulfil institutional requirements, such as the need to publish academic papers and contribute to wider scientific knowledge (Niemczyk and Rónay 2023). As such, a more realistic perception of the use of palaeoecology within UK peatland restoration practice may require insight from the practitioner sphere. This paper aims to further our understanding of the extent and ways in which palaeoecology is utilised in UK peatland restoration practice by attempting to derive insight from a practice perspective. It first does this by examining the characteristics of UK peatland restoration projects. It then looks for evidence for the incorporation of palaeoecology into peatland restoration projects and the reasons why it is or is not considered and incorporated into practice by peatland restoration practitioners.

## 2 Methodology

Data collection to assess the use of palaeoecology in UK peatland restoration projects was completed using two methodological approaches. The first was a review of UK peatland restoration project profiles sourced from the International Union for Conservation of Nature (IUCN) UK Peatland Programme website ‘Projects Map’ (hereafter referred to as IUCNPP‐26). The IUCN UK Peatland Programme is part of the International Union for Conservation of Nature National Committee UK and exists to promote the restoration of UK peatlands through evidence‐based strategy, advocacy, and collaboration between multiple partners including practitioners, policymakers, scientists, land managers and landowners. The purpose of the IUCNPP‐26 review was to look for evidence at project‐scales that palaeoecology had been used or considered as part of the restoration project. IUCNPP‐26 was chosen as it presented a single, easily accessible source of information on UK‐wide peatland restoration projects; however, it is currently closed for review.

As IUCNPP‐26 is not a definitive repository of all UK peatland restoration projects, past and present, the review was supplemented with a self‐guided questionnaire (i.e., one which is completed independently by the respondent) that targeted practitioners within UK peatland restoration projects. The purpose of the survey was to provide insight into a greater number of UK peatland restoration projects and to gain perspective on reasons for the inclusion or exclusion of palaeoecology in UK peatland restoration projects.

### 2.1 Review of IUCNPP‐26

The IUCNPP‐26 review was conducted in 2023. IUCNPP‐26 functioned as a repository of peatland restoration projects, contained as individual profiles for primarily UK‐based projects, though some international projects were present. The research undertaken in this study is part of a larger project considering the value of palaeoecological research for UK peatland restoration practice. Therefore, the ‘map’ function of IUCNPP‐26 was used to include only UK restoration projects, as this is relevant to the focus of the wider project (Figure 1a).

**FIGURE 1:**
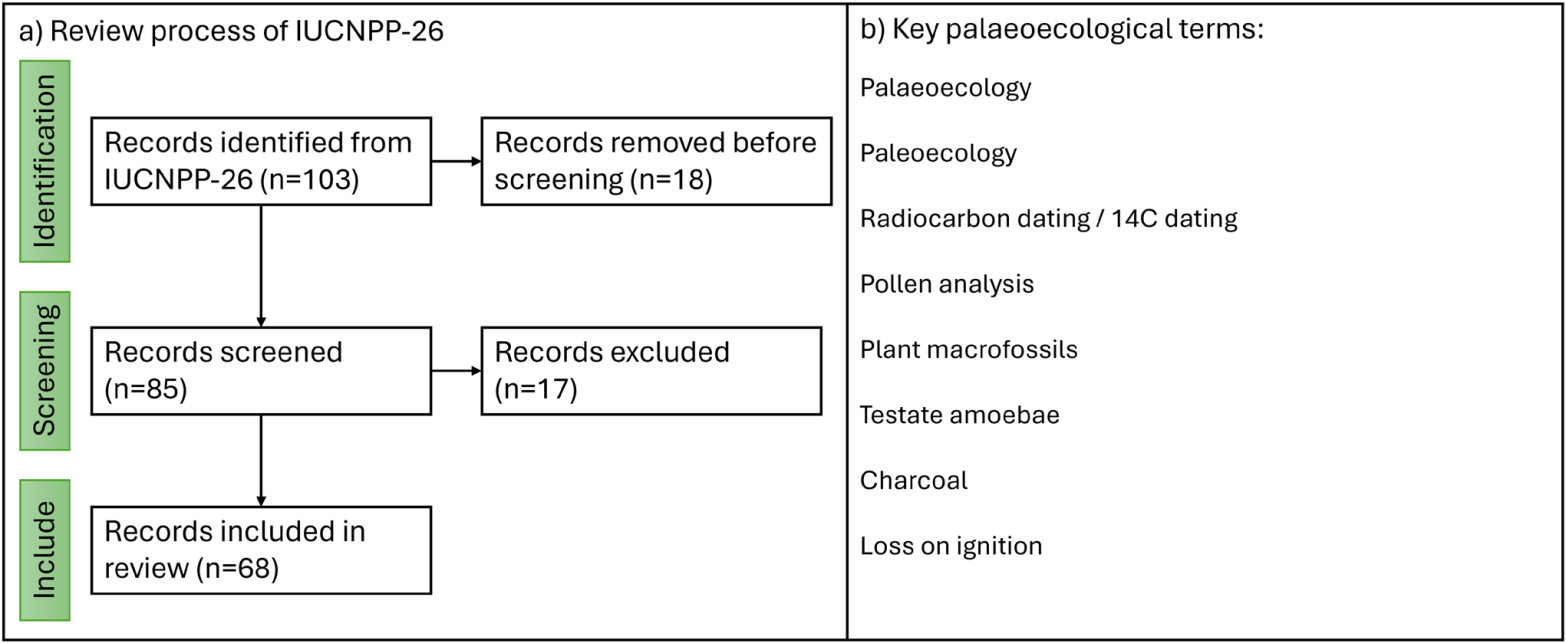
(a) Process of identifying, screening, and including projects sourced from IUCNPP‐26 and the number of records found at each stage. Identified records were removed before screening if they were not UK projects. During screening, records were removed if they did not contain sufficient detail that could support accurate judgement on the inclusion or exclusion of palaeoecology. (b) Key palaeoecological terms used as evidence for the inclusion of palaeoecology, with the inclusion of one or more of these terms accepted as evidence. Terms were guided by a review of common proxies present within palaeoecological studies sourced from Web of Science.

At the time of research, IUCNPP‐26 contained profiles for 85 UK peatland restoration projects, all of which underwent initial review (Figure 1a). Projects were discounted from further analysis if judged to have too little information to make an accurate judgement on the inclusion or exclusion of palaeoecological research. For example, several project profiles did not contain information beyond a project title. Removal of projects with insufficient detail resulted in a final dataset of 68 projects, which were reviewed in more detail.

The IUCNPP‐26 review exercise looked for information to contextualise each project and evidence that palaeoecology had a direct or indirect influence on the restoration project. Information was mined by reading individual project profiles. For project context, the review exercise sought information regarding each project's start date and duration, aims for restoration (e.g., revegetation and listing key species, reference to rewetting and associated mechanisms like drain blocking, or reference to peatland services, such as restoring carbon storage), the restoration techniques employed (e.g., specific restoration methods or activities), and outcomes of the restoration activities (metrics such as number of dams installed or hectares restored, statements regarding target achievement, or comments on success or improvement of conditions), all of which were subject to level of available detail.

Evidence for palaeoecology was found by looking for key palaeoecological terms (Figure 1b). Projects did not have to explicitly use the term ‘paleo/palaeoecology’; instead, links to an associated palaeoecological report, mention of palaeoecological concepts or techniques in the project description, aims, or methods, or partnerships with palaeoecology in a peatland restoration project were identified. Within each of these sources, terms such as ‘pollen analysis’, ‘radiocarbon dating’, ‘plant macrofossil’, ‘charcoal’, or other references to palaeoecological concepts or techniques were accepted as evidence if the term ‘paleo/palaeoecology’ was not explicitly used. These terms were informed by an exploratory literature search of Web of Science to give an overview of commonly employed proxies in peatland palaeoecological studies. The review by Lamentowicz et al. (2025) also confirms these search terms.

### 2.2 Questionnaire of UK Peatland Practitioners

As IUCNPP‐26 relied on project teams uploading or updating the information contained in the project profiles, the level of information available varied between projects. Therefore, additional insight was sought from the UK peatland practitioner community via a predominantly closed question (multiple choice), self‐guided anonymous questionnaire using the survey platform Qualtrics (Supporting Information: Appendix 1).

Questionnaire length and design should consider the research questions and intended analysis and should facilitate response rate through ease of completion (Rowley 2014). Online questionnaires facilitate data collection from many participants, whilst closed questions ensure that the information gathered is relevant to the research focus and, as such, question types are quick to complete, increasing the likelihood that the survey is completed (Jain 2021; Brosius et al. 2022).

The primary aim of the questionnaire was to profile the use of palaeoecology in UK peatland restoration, in line with the overarching research question, rather than understand practitioner attitudes towards palaeoecology; as such, question design focused on characteristics (such as what proxies were investigated) through multiple choice rather than scaled questions (Rowley 2014). Although the use of technical jargon within questionnaires is discouraged (Meadows 2003), due to the methodological focus of the questionnaire (e.g., to gather information on palaeoecological proxies) this was unavoidable. The disadvantages of closed questions (e.g., answers have limited depth, respondents cannot offer answers outside of the available options) were countered with the inclusion of optional open sections which allowed participants to add information that was not captured in the choices that were offered (Meadows 2003).

The structure of the questionnaire largely followed the structure of the IUCNPP‐26 review exercise and focused on gathering contextual information, such as the duration, aims, methods, and outcomes of peatland restoration projects. The length of the questionnaire was kept short, corresponding to lengths advised by, for example, Rowley (2014) to maximise responses and maintain relevance of the questionnaire to the research questions. A questionnaire approach allowed for additional questions that specifically focused on the use, or not, of palaeoecology, the nature of the palaeoecological investigation, and impact on restoration. The questionnaire concluded with an optional open section, allowing respondents to share further information or thoughts not captured in the categorical questions. The questionnaire results were analysed using descriptive statistics to summarise and identify patterns in the data. A participant information sheet provided participants with information on the purpose of the research and data protection and confidentiality. Questionnaire respondents provided electronic informed consent prior to completing the questionnaire.

The questionnaire was promoted through the IUCN UK Peatland Programme website and social media channels from March to November 2024, and during the IUCN UK Peatland Programme annual conference in September 2024. An email campaign was employed for additional survey promotion. Solicitation emails were sent to 21 conservation organisations across the UK. Organisations were chosen on the basis that they demonstrated involvement in peatland conservation (ascertained through organisation websites) and with the aim of achieving a regional overview. Emails were sent to primary contact email addresses sourced from organisation websites. The survey returned 32 responses. As the survey was anonymous, and the number of people to whom the email campaign was forwarded is unknown, the number of non‐responders is unclear.

## 3 Results

### 3.1 Review of IUCNPP‐26

#### 3.1.1 Characteristics of Restoration Projects

The project start dates ranged from 1981 to 2021 with an average project timeframe of 4.6 years (not including projects for which no end date is given). The majority peatland type was blanket bog (37 projects) with the least common type being lowland fen (eight projects).

Six projects did not provide aims and two listed restoration techniques under a ‘project aims’ heading. An overview of the most common aims is summarised in Table 1.

**TABLE 1:** Summary of the most common restoration aims identified from projects sourced from IUCNPP‐26 with similar aims grouped into categories.

Restoration aim	Number of projects
Restore peatland habitat (including ecosystem function, condition and quality and repair/reverse damage)	33
Restore hydrology (including water quality)	23
Revegetating the peatland/restoring target bog species	14

The most common aim amongst restoration projects was to restore peatland habitat, including restoring or improving ecosystem function, condition and quality and repairing or reversing damage. Many projects that included revegetation as a restoration aim gave specific target species, such as cotton grass or *Sphagnum*; however, some projects more generically stated the restoration of ‘bog vegetation’ as the revegetation target or as an expected outcome of the restoration. 21 projects included aims beyond direct restoration of peatland ecosystems, such as: socio‐economic benefits for the wider community (such as education and recreation, five projects); aims to increase carbon storage or reduce carbon emissions (nine projects); supporting and collaborating with local landowners and farmers, including supporting grazing (two projects) and resolving disputes over land use (one project); and supporting the provision of ecosystem services (six projects), including the development and delivery of Payment for Ecosystem Services schemes (two projects).

An overview of the most common restoration techniques is summarised in Table 2. Most projects included methods to restore or manage hydrology, although less than half of the reviewed projects included hydrological restoration as a specific project aim. Several projects referenced the use of grazing as a method for managing excess or undesirable vegetation; however, two projects listed the reduction of grazing as part of the restoration techniques, with ten projects in total mentioning grazing in some capacity. Similarly, several projects included tree and/or scrub removal as a restoration method, yet two projects included tree planting or the identification of areas suitable for tree planting within the project methods. Three projects did not detail restoration techniques.

**TABLE 2:** Summary of the most common restoration techniques identified from projects sourced from IUCNPP‐26 with similar methods grouped into categories.

Restoration method	Number of projects
Hydrological management (drain/gully blocking, bund creation, dam installation)	53
Tree/scrub removal	27
Revegetation (plug planting, *Sphagnum* propagation)	17
Vegetation management (*Molinia* removal, invasive species management)	15
Of which include grazing	8

#### 3.1.2 Evidence of Palaeoecology Within Restoration Projects

An overview of the evidence for inclusion of palaeoecology within restoration projects is summarised in Table 3. Most projects did not evidence the inclusion of palaeoecology. Two projects gave direct evidence that palaeoecological research was undertaken to support the peatland restoration; this included a report by Fyfe et al. (2008) which presents the outcome of palaeoecological research commissioned by Exmoor National Park Authority. Three projects gave indirect evidence for the inclusion of palaeoecology in the restoration project. Indirect evidence included reference to palaeoecological methods on the project or conservation agency's website, with no further detail on how the project intended to make use of the results to support site restoration.

**TABLE 3:** Summary of evidence for inclusion of palaeoecology in UK peatland restoration projects identified from projects sourced from IUCNPP‐26.

Evidence for palaeoecology	Number of projects	Example of evidence
No	63	—
Direct evidence	2	Palaeoecological report to support archaeological evaluation of the restoration site
Indirect evidence	3	Peat core recovered from blanket bog for pollen analysis to inform woodland replanting

Contextual factors for the five projects that evidenced palaeoecology reflected the wider trends summarised in Tables 1 and 2. Project aims were variable, with a focus on restoring peatland quality and function and prominence of hydrological restoration techniques within project methods. Both projects that gave direct evidence of palaeoecology focused on blanket bog. Three projects did not give an end date, and two projects lasted for 5 and 8 years respectively.

### 3.2 Survey of UK Peatland Practitioners

Of the 32 survey responses, there was one direct (same project) and one indirect (same organisation, different restoration project) overlap with the projects evaluated within the IUCN website review. An overview of the use of palaeoecology reported by the survey respondents is presented in Table 4. Reasons for not including palaeoecology in peatland restoration projects are summarised in Table 5.

**TABLE 4:** Summary of reported use of palaeoecology in UK peatland restoration projects from questionnaire of UK peatland practitioners.

Use of palaeoecology	Number of projects
Yes	7
No	24
Unsure	1

**TABLE 5:** Summary of reported reasons for not including palaeoecological research in UK peatland restoration projects from questionnaire of UK peatland practitioners.

Reason	Number of projects
Lack of time	10
Lack of funding	13
Lack of expertise/academic input	12
It is not a priority	9
Other	8

Table 4 shows that most survey respondents reported that palaeoecological research was not used to inform peatland restoration. Table 5 shows that a lack of funding was the most common reason for not including palaeoecological research within peatland restoration projects (13 projects), closely followed by a lack of expertise/academic input (12 projects). Palaeoecology not being a priority was the least selected reason (nine projects).

Of the eight responses that did report use of palaeoecology (including one unsure), four indicated that doing so directly informed the restoration. Only one response stated that the palaeoecology did not impact the restoration, due to insufficient funding. The results and/or use of the palaeoecological research were most commonly reported in a project report or grey literature (five projects), with only one project publishing its findings in an academic paper. In three cases, further information on the incorporation of palaeoecology was found through a Google search (e.g., through searching the project name with ‘palaeoecology’, in addition to terms such as ‘pollen’ or ‘radiocarbon dating’). This search returned two project websites and one visitor leaflet. Although none of these resources used the term ‘palaeoecology’, each had clear reference to palaeoecological methods or understanding the past environment; for example, two of the resources highlight pollen analysis as a means of understanding past vegetation. Only one of these three projects stated that the palaeoecology was used to directly inform the restoration work; however, direct impact (such as using palaeoecology to define baseline site conditions or inform restoration targets) was not evident in the found resource related to this project.

An overview of the palaeoecological methods employed in the projects is outlined in Table 6 and the involvement of practitioners in the research is outlined in Table 7.

**TABLE 6:** Summary of reported palaeoecological proxies used in UK peatland restoration projects from questionnaire of UK peatland practitioners.

Palaeoecological method	Number of projects
Pollen	7
Plant macrofossils	3
Testate amoebae	2
Radiocarbon dating	7
Loss on ignition	2
Charcoal	3
Other	1

**TABLE 7:** Summary of reported practitioner involvement in the research process from questionnaire of UK peatland practitioners.

Research stage	Number of projects
Defining research questions	4
Fieldwork	8
Generating palaeoecological data	2
Processing palaeoecological data	3
Interpreting results	5
Reporting of palaeoecological data	3
Other	0

Table 6 shows the survey respondents reported pollen and radiocarbon dating to be the most employed proxies in palaeoecological studies undertaken to inform peatland restoration (seven projects). Plant macrofossils and charcoal were reported an equal number of times (three projects), with testate amoebae and loss on ignition each appearing the least in the survey responses (two projects). No other proxies were reported in the open section of the related question.

Table 7 shows that survey respondents reported that practitioners were most involved in the fieldwork stage of the research process compared to other research activities. Practitioners were more likely to be involved in early stages of the research, that is, defining research questions (four projects) and fieldwork (eight projects), compared to later stages of the research, that is, interpreting results (five projects) and reporting palaeoecological data (three projects).

## 4 Discussion

### 4.1 Is Palaeoecology Utilised in UK Peatland Restoration Practice?

The underutilisation versus potential of palaeoecology to inform wider conservation practice has become a much‐discussed topic over the last 30 years (J. J. B. Birks 1996; H. J. B. Birks 2012; Froyd and Willis 2008; Davies and Bunting 2010; Davies 2011; Manzano et al. 2020; Siggery et al. 2023). As such, this study's findings that palaeoecology appears to have been rarely utilised within UK peatland restoration practice is unsurprising. The logistical reasons gathered from the survey for the underuse of palaeoecology in UK peatland restoration, namely lack of funding and time, are consistent with barriers to applied palaeoecology identified in previous research (e.g., Gauld et al. 2025). Lack of funding aligns with wider issues within peatland conservation—for example, one respondent highlighted how ‘we'd love palaeoecological data, but funding is only for restoration works’. This could be reflective of the broader structure of managing restoration projects, where limitations in funding can affect evidence‐based conservation actions or monitoring and evaluation activities (Al‐Fulaij et al. 2025). Within a funding‐led model of conservation, there often is not scope (in terms of time, money, and other resources) for elements that do not have immediate, perceivable payoff; this means that activities that have longer‐term or less instrumental benefits can be less prioritised by funders and practitioners (Gauld et al. 2025).

Yet the funding landscape for UK peatland restoration is changing. Increasing amounts of private financing may bring opportunities for activities that are typically less prioritised or unfeasible due to limitations in public funding (Moxey et al. 2021; Artz et al. 2023). In tandem with this funding shift is a greater consideration of the historic environment, including the palaeoenvironmental record, within more recently published strategies for peatland conservation (e.g., *England Peat Action Plan*; DEFRA 2021) in response to advocation from the academic community (e.g., Fyfe et al. 2019) and several years of cross‐sector collaboration (Hazell et al. 2022). Subsequent guidance from Historic England and Natural England recognises both the importance of protecting historic environment features (including archaeological features, cultural artefacts, and palaeoenvironmental remains) through well‐planned restoration work and the value of palaeoenvironmental or historical records for informing site restoration (Historic England and Natural England 2022; Historic England 2025). Reference to peatland heritage and palaeoecology is also present in the latest Peatland Code guidance, a voluntary standard to support private funding of peatland restoration (IUCN UK Peatland Programme Peatland Code 2.1 Guidance 2024).

The underuse of palaeoecology in UK peatland restoration thus far may reflect the lack of consideration for the historic environment in previous guidelines for peatland conservation and broader marginalisation of palaeoecological perspectives from conservation policy (Davies and Bunting 2010). Consideration of the historic environment within the most recent guidance and standards for peatland restoration represents an important shift in the space for palaeoenvironmental and archaeological work within peatland restoration, with Historic England (2025) recommending collaboration between natural and historic environment stakeholders from the outset. However, this and other studies (e.g., Siggery et al. 2023; Gauld et al. 2025) highlight that funding is often not available within peatland restoration projects for palaeoecological study, even when practitioners are aware of palaeoecology and appreciative of its value for restoration. This is exemplified in England's Peatland Restoration Roadmap Report (Natural England 2026, Annex 3, Case Study 8) which highlights the value of palaeoecology for guiding future management and supporting wider community engagement but also notes the need for alternative funding to achieve this work. Furthermore, this recognition of the historic environment is not equal across all UK peatland standards, with little mention in the current draft of Scotland's Peatland Standard (NatureScot 2026). Although the increasing recognition of the historic environment within peatland guidance and standards may lead to greater provision for palaeoecology as part of peatland restoration strategy, palaeoecology is not yet embedded as a concept within broader technical guidance and standards.

This study indicates that logistical barriers are balanced with a lack of expertise and academic input as reasons for a lack of palaeoecological insight. Some questionnaire respondents expressed limitations in knowledge of palaeoecology and its value for conservation. For example, one respondent stated that they ‘*do not know what palaeoecological research is and how it would help the project*’ whilst another stated that ‘*the benefits of using the research not [sic] appreciated or elucidated*’. This is synonymous with the findings of Siggery et al. (2023), which illustrated that experience with palaeoecology is not widespread amongst the practitioner community.

A lack of expertise or academic input coupled with a lack of understanding highlights the need for continued promotion of palaeoecology in relevant practitioner spheres. Efforts to enhance visibility and accessibility of palaeoecology in conservation practice have increased in recent years, including collaborative, practice‐focused approaches to palaeoecology published in open‐access journals and, in some cases, accompanied by plain‐language blog posts on conservation organisation websites (McCarroll et al. 2016a; Siggery et al. 2025). Such efforts indicate that positive steps are being taken for the integration of palaeoecology in conservation practice. Establishing working relationships between academic and conservation organisations is an essential step; however, breakdowns in these partnerships can occur once the project or funding ends or if staff turnover results in loss of institutional knowledge (Mearns 2012). Attendance and contribution to practitioner‐focused conferences and webinars would support broader outreach and promotion of palaeoecology for peatland restoration, though this may be challenging to balance with attendance at academic conferences.

### 4.2 Is There Consistent Reporting of the Use of Palaeoecology in UK Peatland Restoration?

This study indicates that, from the perspective of practice, there are few concrete examples of applied palaeoecology within UK peatland restoration, suggesting that UK peatland restoration projects do not regularly engage with palaeoecology. However, this finding may also indicate that palaeoecological research is not clearly documented in the practitioner sphere. One of the few examples evident through IUCNPP‐26 relates to the work of McCarroll et al. (2016a, 2016b, 2017) in partnership with Yorkshire Peat Partnership (YPP). This research, including the associated PhD thesis, is referenced in Thom and Hinchley's (2019) report on 10 years of restoring Yorkshire's upland peatlands. Additionally, McCarroll et al. (2016a) highlights that the palaeoecological research was used by YPP to secure funding that would enable the continuation of experimental Sphagnum translocation studies. The acknowledgment of the research within the practitioner report, the specific use of the research to support future restoration through securing funding, and the reference to collaborative research methods within the associated papers (e.g., joint site selection and formulation of research questions with practitioners) illustrates a unique bridge across palaeoecological research and restoration practice that is not replicated across other examples of applied palaeoecology.

Internationally, palaeoecology has a substantial history of application and potential application in conservation, though resulting changes in conservation practice are less easy to trace from academic literature (Wingard et al. 2017; Bunting et al. 2026). Clear links between palaeoecology and international conservation efforts are seen in the management of the Everglades wetlands in the USA, where palaeoecological and palaeoenvironmental data contributed to setting reference conditions within the Comprehensive Everglades Restoration Plan (Riedinger‐Whitmore 2016), and in conservation efforts in the Galápagos islands, where palaeoecology distinguished between species that were actually native and ecological targets that were reflective of modified systems (Bunting et al. 2026). A key similarity between these success stories from the wider global context and the UK work of McCarroll et al. (2016a, 2016b, 2017) and related works of Chambers et al. (1999, 2013, 2017), Chambers, Mauquoy, Cloutman, et al. (2007) and Chambers, Mauquoy, Gent, et al. (2007) is that they are the result of many years of collaboration between researchers, practitioners, and central and local government agencies. The influence of evolving, multi‐stakeholder collaborations on conservation may not be readily captured in reports or academic literature, particularly where contribution lies in informing discussions, building relationships, and changing perceptions rather than in changing management approaches (Bunting et al. 2026).

It follows, therefore, that the use of palaeoecology in peatland restoration is more evident through surveying practitioners than through available information on peatland restoration projects. For ongoing projects, the absence of a project report does not necessarily reflect the status of research uptake or impact on practice, as it is possible that such materials have not yet been produced. However, it is also possible that the lack of easily accessible evidence for palaeoecology within peatland restoration is reflective of broader reporting conventions and lack of consistent documentation within conservation practice (Sutherland et al. 2004; Dubois et al. 2020). There is an acknowledged need for greater reporting, or recording, of conservation practice, which is often based on accumulated experience rather than published information (Sutherland et al. 2004; Fabian et al. 2019). Although tacit knowledge and anecdotal experience can be valuable in guiding approaches to conservation (Fazey et al. 2006), it is often not collected in easily transferrable or distributable forms (Sutherland et al. 2004).

### 4.3 Increasing the Visibility of Palaeoecology in UK Peatland Restoration Practice

The lack of clear examples found through this study supports the need for greater documentation of palaeoecology within peatland restoration practice. Formatting examples as case studies would address this gap and support wider appreciation of palaeoecology amongst practitioners, as case studies can help to contextualise the relevance of the research in real‐world situations (Lavery et al. 2021; Siggery et al. 2023). The PAGES special issue *Using Paleoecology for Restoration Ecology* presents several such case studies that highlight where palaeoecology could contribute towards restoration ecology, including challenging assumptions regarding natural baseline species in New Zealand (Nogué et al. 2022) and guiding the use of prescribed ground fires to manage Western Mediterranean *Pinus nigra* forests (Morales‐Molino and Schwörer 2022).

Greater documentation of palaeoecology in practice could be achieved with greater involvement of practitioners at the reporting stage. It is encouraging that the questionnaire indicated practitioner input in multiple stages of the research process, although the responses indicate that practitioners are more likely to be involved in earlier stages of the research process (defining research aims, fieldwork) than in later stages (interpreting results, reporting results). This is understandable, as interpreting and presenting palaeoecological data may seem to be outside of practitioner expertise compared to fieldwork, which can be more in line with practitioner ways of working (Gauld et al. 2025). However, incorporating the contextual and experiential knowledge of practitioners into result interpretation can relate findings to management priorities and ensure that recommendations are achievable (Gauld et al. 2025). For example, Siggery et al. (2025) demonstrate how collaboration with Surrey Wildlife Trust helped align palaeoecological research and resulting management recommendations with practitioner priorities and obligations, such as ensuring recommendations for experimental *Molinia* management did not conflict with existing frameworks. Further to this, greater practitioner involvement in reporting and disseminating research could help make the contribution of palaeoecology to restoration more explicit and provide a clearer record of its practical application.

There have been calls for an evidence‐based approach to conservation form the early 2000s in response to the acknowledged difficulties of a predominantly experience‐driven knowledge base (Pullin and Knight 2001; Sutherland et al. 2004; Fabian et al. 2019; Dubois et al. 2020). Uptake of this approach is evident. For example, the Conservation Evidence project (www.conservationevidence.com), based at the University of Cambridge, offers free summaries of scientific literature, with some conservation organisations (e.g., Essex Wildlife Trust) developing dedicated webpages which showcase their commitment to evidence‐based conservation. This resource is supported with a freely available Evidence Toolkit (Smith et al. 2023), which provides resources for academics, practitioners, and other decision‐makers to facilitate greater use of evidence in conservation (Smith et al. 2023). At time of writing, searching the Conservation Evidence website using the terms ‘palaeoecology’ or ‘paleoecology’ does not return any summaries. This highlights both a gap and a potential, as palaeoecologists could engage with this resource and the toolkit to support more effective engagement with practitioners and policymakers and promote palaeoecology in accessible and practice‐focused format to a wider audience. This format could follow the structure of studies within Conservation Evidence, which include a one‐sentence action summary and plain language project synopsis.

The development of a similar centralised and searchable resource platform for palaeoecology and better facilitation of communication between and amongst science and practice (e.g., through boundary organisations, which bridge the space between research and practice, Cash et al. 2002) could improve information transfer between palaeoecologists and practitioners (Farwig et al. 2017; Fabian et al. 2019). This platform would be particularly useful if it learned from Conservation Evidence, whereby project profiles are fronted by practice‐focused overviews. Achieving this would require a collaborative effort between academic and practitioner organisations to design and maintain a usable database that is driven by practitioner needs but guided by palaeoecological expertise to translate research into understandable formats (Gillson and Marchant 2014). This concept is evident in the PeatDataHub (peatdatahub.net), a central repository for peatland research papers designed to support a network for collaboration and knowledge exchange. However, uptake, and therefore content, of the PeatDataHub are limited as such platforms require long‐term funding support and adequate resources to continually update, promote, and embed them amongst organisations.

### 4.4 The Nature and Place of Applied Palaeoecology in UK Peatland Restoration

Although this study only returned a few examples where palaeoecology was used, several similarities can be drawn between these projects. The 8 examples returned by the questionnaire all involved a partnership with an academic organisation; however, only one project stated that the palaeoecology was undertaken as part of academic research or student projects, stating that the ‘*data was collected by different students over a long period of time* *’*. The range of funding sources illustrates that the palaeoecology was not entirely dependent on academic‐driven funding. Management‐focused funding sources included the Nature for Climate Peatland Grant Scheme and Welsh Government Sustainable Management Scheme. Pollen and radiocarbon dating were the most common proxies, with only one project stating the use of existing palaeoecological data.

The open responses of the questionnaire indicate a misalignment between what palaeoecology can offer and what has been prioritised in UK peatland restoration practice. One participant captured this in sharing: ‘*If there were grants and expertise available to assist, I'm sure a lot of people would love to do this! It doesn't relate directly to restoration efforts (i.e., the blanket “hectares restored” that we all have to work towards), so the focus isn't on this at the moment*’. A second participant highlighted how time required for research may not be reflected in the added value, stating that they were: ‘*unsure of the benefits* [of palaeoecology] *to the project. If it will aid in delivering the best restoration possible then it is worthwhile, however if it slows down restoration without benefit then we don't have time if we are to reach 2 million ha by 2040*’. These quotes are indicative of wider factors within peatland restoration (i.e., a target of ‘hectares restored’ within a certain timeframe) that may influence the relevance and value of palaeoecology to individual restoration projects.

There is a rich discussion in the literature regarding the knowledge that conservation practice might access through palaeoecological research (Froyd and Willis 2008; Clarke and Lynch 2016; H. J. B. Birks 2019; Goodenough and Webb 2022); however, palaeoecology is not equally relevant to all aspects of peatland restoration. As an example, management of UK blanket peatlands often requires stabilisation of eroding peat as a priority to prevent further development of erosional landforms, such as gullies, and negative impacts on ecosystem services such as carbon storage and water quality (Andersen et al. 2017). Research into solutions, such as stabilising through revegetation and/or geotextiles (Watts 2020) or the effectiveness of stabilisation strategies (Shuttleworth et al. 2015) directly inform immediate management priorities. In such contexts, palaeoecology may be less relevant as it does not prevent further degradation or contribute to the short‐term, observable restoration outcomes commonly prioritised by conservation funders (Gauld et al. 2025). This does not diminish the value of palaeoecology but highlights the need to consider its contribution in relation to the objectives and timeframes of individual restoration projects.

However, the most common target highlighted in the IUCNPP‐26 review was to restore peatland habitat, encompassing ecosystem function, condition, and quality. Establishing such targets needs clear and evidence‐backed understanding of what the restoration is working towards and whether that goal is achievable (Taylor et al. 2019). Palaeoecology could contribute to this evidence base by revealing how historical environmental conditions and land use have shaped ecosystems, thereby helping to identify ecologically suitable and achievable restoration targets. The consequences of overlooking ecological history are illustrated by mangrove restoration in the Philippines, where planting undertaken without sufficient consideration of past ecology has resulted in high failure rates and expensive and damaging outcomes for existing ecosystems (Samson and Rollon 2008; Taylor et al. 2019).

In peatlands, palaeoecology can similarly inform management by placing contemporary conditions within the context of historic disturbance and land‐use. Woodbridge et al. (2026) illustrate variable fire and landscape dynamics through time, with some sites displaying minimal long‐term changes in vegetation and carbon accumulation following episodic fires, and other sites failing to recover pre‐fire conditions. This suggests that palaeoecology could guide management strategies and priorities to help account for differences in site vulnerability within management strategies, particularly as wildfire risk may increase with climate change (Woodbridge et al. 2026). Similarly, testate amoebae‐based hydrological reconstructions have demonstrated a widespread drying of European peatlands, compared to historical baselines (Swindles et al. 2016). Swindles et al. (2016) highlight that such hydrological shifts, and their potential to trigger irrecoverable changes in vegetation and the long‐term stability of carbon stores, should be considered within restoration strategies. Together, these studies illustrate how palaeoecology can inform management decisions where long‐term ecological changes and responses to historic disturbances are relevant to restoration objectives and contemporary contexts.

Still, the application of palaeoecology must be considered alongside the practical priorities and resources of the restoration. Andersen et al. (2017) identified ditch and drain blocking as the most widely implemented restoration activities across western Europe, a pattern that is also reflected in the IUCNPP‐26 review, where hydrological management featured in 53 projects compared to revegetation in 17 projects. Although these interventions may benefit from a historical perspective, particularly considering the widespread shift towards drier conditions evident in Swindles et al. (2016), their immediate implementation does not require site‐specific palaeoecological investigation. The contribution of palaeoecology may therefore extend beyond individual interventions to informing national and international restoration priorities, including contextualising contemporary environmental conditions, identifying vulnerable systems, and guiding restoration targets and measures of success.

There may be a tendency to only focus on how palaeoecology can directly and impact restoration. In reality, it is rare that the impact of research on conservation practice happens quickly or is easily traceable (Lawson et al. 2017; Djenontin and Meadow 2018). Moreover, impact does not need to be instrumental (i.e., direct use of knowledge to change or inform practice, Waylen and Young 2014; Edwards and Meagher 2020) in order to be valuable. In some cases, palaeoecology has a more conceptual or substantive impact on peatland restoration, by encouraging and providing alternative perspective on the landscape. For example, palaeoecological analysis has been used to challenge the assumed antiquity of vegetation in both island (Bush et al. 2014) and peatland (McCarroll et al. 2016a, 2016b) environments, illustrating that restoration targets may reflect a modified system than the longer‐term ecological history of the site. In the survey results, the value of the palaeoecological research appeared to lay more in its ability to tell a story of landscape evolution and support the securement of further fundings that in its potential to directly inform restoration (McCarroll et al. 2016a, 2016b, 2017). This application of palaeoecology is acknowledged within archaeological research, where palaeoecology can elucidate historical human‐environment interactions and the development of cultural landscapes and support cultural heritage management (Hjelle et al. 2012; Mayle and Iriarte 2014).

Considering the wider potential impact of palaeoecology on UK peatland restoration impact has two implications. The first implication is that less tangible impact, such as changing perceptions or understanding of the landscape, is less likely to be captured in restoration outcomes and reports. Purely focusing on published outputs may give a false perspective on the status of palaeoecology in peatland restoration. This is exemplified by this research, as the questionnaire indicates a greater presence of palaeoecology in practice than could be found through IUCNPP‐26, although the small sample size of the study is acknowledged. The second implication is that the applied value of palaeoecology for peatland restoration practice may be expanded when it is not considered in isolation (Andersen et al. 2017; Goodenough and Webb 2022). Decision‐making in conservation is often based on several factors (Djenontin and Meadow 2018). Therefore, opportunities to improve the visibility and accessibility of palaeoecological evidence through evidence‐sharing platforms, such as the Conservation Evidence database and PeatDataHub, could help to integrate a longer‐term perspective into a holistic evidence base for peatland restoration.

On the basis of these findings, this study makes two recommendations to support the application of palaeoecological research in UK peatland restoration practice. The first is for better dissemination and documentation of case studies to provide greater evidence of how palaeoecology may be used to support UK peatland restoration and clearer insight into the use of palaeoecology in practice. This may be facilitated by using existing platforms and jointly sharing reporting where possible between practitioners and palaeoecologists. The second recommendation is for better two‐way knowledge transfer and greater collaboration between palaeoecologists and practitioners to identify restoration challenges and knowledge gaps where palaeoecology could contribute and to ensure that research is in line with management aims and resources.

## Author Contributions

**J. Gauld:** conceptualization (lead), data curation (lead), formal analysis (lead), methodology (lead), writing – original draft (lead), writing – review and editing (equal). **E. L. Shuttleworth:** supervision (equal), writing – review and editing (equal). **W. Fletcher:** supervision (equal), writing – review and editing (equal). **J. Akerman:** methodology (supporting), writing – review and editing (equal). **H. Harper:** methodology (supporting), writing – review and editing (equal).

## Funding

The authors have nothing to report.

## Conflicts of Interest

The authors declare no conflicts of interest.

## Supporting information


**Supporting Information Appendix 1:** Self‐guided anonymous survey of UK Peatland Practitioners hosted on Qualtrics: Full questionnaire.

Supporting Information Data 1:

## Data Availability

Data is provided as Supporting Information.
