# Supplementary material for: The Use of Palaeoecology in UK Peatland Restoration: Perspectives From Practice
Source: Ecol Evol. 2026 Nov 13;16(11):e74620. doi: 10.1002/ece3.74620 (PMC13768584; doi:10.1002/ece3.74620)
Supplement: Supplementary file 1 [file ECE3-16-e74620-s002.docx]

Appendices

Appendix 1. Self-guided anonymous survey of UK Peatland Practitioners hosted on Qualtrics: Full questionnaire

Q1. – Please provide the name and duration of the peatland restoration project [written answer]

Q2. – Was palaeoecological data used to inform or support the peatland restoration project? [checklist]

Yes / No / Unsure

**If yes/unsure – survey moves to Q3a and then continues with the rest of the survey.**

**If no – survey moves to Q3b and ends here.**

Q3a. – What palaeoecological proxies were investigated? [checklist]

Pollen.

Plant macrofossils.

Loss on ignition.

Charcoal.

Testate amoebae.

Radiocarbon dating.

X-ray fluorescence (XRF)

Other [specify]

Q3b. – For what reason(s) was palaeoecological research not used to inform the restoration project? [checklist]

Lack of time.

Lack of funding.

Lack of expertise/academic input.

It is not a priority.

Other [expand].

Q4. – How was the palaeoecological research generated? [checklist]

Expertise within the practitioner team

Partnership with academic organization

Other [expand]

Q5. – How was the research funded? [short written answer]

Q6. – Were practitioners involved in any of the following research stages? [checklist]

Defining research questions.

Fieldwork.

Generating data (example: processing samples, identification of material).

Processing data.

Interpreting results.

Reporting of palaeoecological data.

Other [expand]

Practitioners were not involved in the research process.

Q7. – How did the palaeoecological research inform the restoration work? [checklist]

The palaeoecological data directly informed the restoration work (example: the data informed the restoration targets for the study site).

The palaeoecological data indirectly informed the restoration work (example: the data was used to support funding bids).

The palaeoecological data did not impact or inform the restoration work.

Other [expand]

**If ‘It did not impact the restoration work’ is selected**:

Q7b. For what reason(s) did the palaeoecological research not impact the restoration work? [checklist]

Data was not relevant to the restoration work.

Recommendations from the palaeoecological data could not be implemented due to lack of time.

Recommendations from the palaeoecological data could not be implemented due to lack of funding.

The implications of the palaeoecological data for practical restoration were unclear.

Other [expand]

Q8. – Was the use of palaeoecological data reported in any of the following: [checklist]

Restoration project webpage.

Project report/grey literature.

Academic paper.

IUCN peatland programme project map.

Presented at academic conference or meetings.

Presented at practitioner conference or meetings.

The use of palaeoecological data was not reported.

Other [expand]

**If ‘not reported’ is selected:**

Q8b. For what reason(s) was the use of palaeoecological data not reported?

It was more important to highlight other aspects of the restoration work.

The palaeoecological data was not relevant or useful to the project.

Lack of expertise in presenting or explaining the palaeoecological work.

Other [expand]

Q9. (optional) Any other comments [written answer]
